# Analysis of Multiclass Antibiotics in Lettuce by Liquid Chromatography–Tandem Mass Spectrometry to Monitor Their Plant Uptake

**DOI:** 10.3390/molecules24224066

**Published:** 2019-11-10

**Authors:** Beatriz Albero, José L. Tadeo, María del Mar Delgado, Esther Miguel, Rosa Ana Pérez

**Affiliations:** Departamento de Medio Ambiente y Agronomía, Instituto Nacional de Investigación y Tecnología Agraria y Alimentaria (INIA), Ctra. de la Coruña, Km. 7, 28040 Madrid, Spain; albero@inia.es (B.A.); tadeo@inia.es (J.L.T.); delgado@inia.es (M.d.M.D.); emiguelg@inia.es (E.M.)

**Keywords:** pharmaceuticals, veterinary antibiotics, ultrasonic assisted extraction (UAE), dispersive solid-phase extraction (dSPE), compost, lettuce, liquid chromatography–tandem mass spectrometry

## Abstract

The main entry routes of antibiotics in the environment are the application of organic wastes to improve soil quality and the irrigation with recycled water. Once in the environment, antibiotics can be introduced in the food chain through their uptake by crops. This paper describes the development of an analytical method based on ultrasound-assisted extraction for the determination of seven antibiotics in lettuce. The developed method was applied to evaluate antibiotic uptake by lettuce grown in pots fertilized with composted poultry litter doped with a mixture of antibiotics to reach a final concentration of 2.5 µg/g in soil. Lettuce were harvested after 21, 36, and 55 days. Five of the seven studied antibiotics were found in all samples. The highest uptake was found for lincomycin (51 ng/g fresh weight) followed by sulfamethoxazole (44 ng/g fresh weight) and sulfamethazine (21 ng/g fresh weight) in lettuce harvested after 21 days. An important decrease of their levels was observed after 36 days, but these levels remained similar after 55 days. Although levels found in lettuce were low, the presence of antibiotics demonstrates the need for further assessing food safety risks related with the use of soil amendments or irrigation water contaminated with antibiotics.

## 1. Introduction

The intensive use of antibiotics to prevent or treat bacterial infections has led to the rapid expansion of antibiotic resistance that threatens the efficacy of the treatment of infectious diseases in both humans and animals. Antibiotics are not completely metabolized in the body and a significant fraction administered to humans and animals (between 17% and 90%) is excreted unchanged [1,2]. The main routes of entry of these compounds into the environment are the application of sewage sludge, biosolids and manure as soil amendment or fertilizers, and the use of recycled water for irrigation [1,2,3]. Once antibiotics are released into the environment, their behavior and uptake by plants depends not only on their physicochemical properties, but also on the characteristics of soil, the crop and its growing conditions. Root uptake is expected to be an important route of plant exposure to antibiotics, being these compounds detected in fruits and other edible parts of crops due to their translocation from root to aerial tissues. Recently, a review by Pan and Chu [4] provided an overview of the fate of antibiotics in soil and their uptake by edible crops including their concentration values in different crops. In general, lettuce, radishes, and carrots are the most studied edible vegetables because they are selected as models of raw leaf and root vegetables commonly consumed. Boxall et al. reported in 2006 the potential of antibiotics to be taken up from soil by plants for human consumption and their significance to human health [5]. Their results demonstrated that veterinary medicines applied to soils at environmentally realistic concentrations were taken up at detectable levels. Consequently, greater attention was then paid to determine levels of antibiotics in plants and to evaluate the uptake of antibiotics by plants from fertilized soils or soil spiked with antibiotics [5,6,7,8,9,10,11,12], irrigated with water contaminated with antibiotics [13,14,15,16] or treated wastewater [17,18,19].

The methods for the determination of pharmaceutical and personal care products in plant tissues were recently reviewed by Wu et al. [20], being traditional solid–liquid extraction (SLE), ultrasonic assisted extraction (UAE), and pressurized liquid extraction (PLE) the main extraction techniques used. After extraction, a cleaning step using SPE cartridges was usually performed. In the study developed by Chitescu et al. [6], two extraction techniques, PLE and UAE, were compared for the determination of forty-two compounds (pharmaceuticals, azole biocides, and fungicides) and the extracts were purified and concentrated by applying SPE. In this work, the effects of different extraction solvents and extraction conditions were tested; and, although both extraction approaches could be applicable, the optimal extraction efficiency was obtained by PLE using solvent mixtures containing acetone for soil and methanol for plant samples. Nevertheless, the highest recoveries reported for some of the antibiotics studied were ≤60%. In order to have quick and effective methods to extract pharmaceuticals from vegetables, Hu et al. [21] and Chuang et al. [22] reported two different QuEChERS (Quick, Easy, Cheap, Effective, Rugged and Safe) methods. Thus, Hu et al. [21] developed a method for the extraction of 26 veterinary antibiotics from vegetables where samples were extracted with acetonitrile–methanol (85:15, *v*/*v*) and citric buffer solution, followed by liquid–liquid partitioning adding anhydrous magnesium sulfate and sodium chloride. In general, recoveries in the range of 60–98% were obtained, except for sulfaquinoxaline, sulfaclozine, and doxycycline with average recoveries of 46%, 54%, and 58%, respectively. In a study performed by Sallach et al. [23] four methods for the extraction of four antibiotics of different classes from lettuce were compared. In this work, the extraction methods evaluated included freeze-and-thaw cell lysing, mechanical maceration, ultrasonic tissue homogenization, and microwave-assisted extraction. Freeze-and-thaw cell lysing provided the highest extraction efficiency with recoveries from 44% to 139%. Recently, Tadic et al. [24] developed an UAE method for the determination of 10 antibiotics and six of their metabolites in four vegetable matrices but for some of the target compounds the relative recovery results were still <60%. As part of the validation procedure, the analytical method was applied to the analysis of lettuce, tomato, broad beans and cauliflower grown in peri-urban areas that were irrigated with reclaimed waters. Two of the studied antibiotics, ofloxacin and sulfamethoxazole, were detected in all the samples analyzed and their highest levels were found in lettuce leaves. The results obtained evidenced the transfer of antibiotics from the environment into crops.

The multiresidue analysis of antibiotics in vegetables presents many difficulties due to the complexity of the matrix and the different physicochemical properties of these compounds. The aim of this study was to develop a selective, sensitive, and efficient analytical method for quantitative determination of seven representative antibiotics belonging to different classes (fluoroquinolones, tetracyclines, sulfonamides, and lincosamides) in lettuce. The validated method was applied to determine the uptake of these compounds by lettuce grown in a soil–manure system.

## 2. Results

### 2.1. Sample Preparation

**Figure 1:**
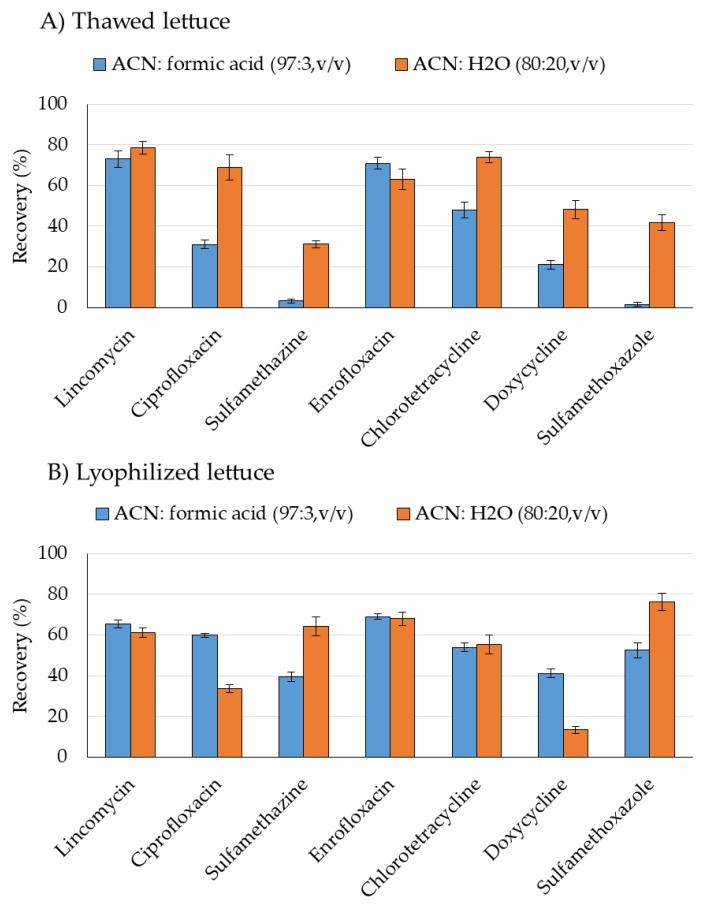
Influence of the extraction solvent on the recovery of 200 ng of each antibiotic spiked in (**A**) thawed lettuce and (**B**) lyophilized lettuce. Each assay was performed with three replicates.

UAE was selected because it provides an exhaustive extraction with a low consumption of solvents and short extraction time due to its increased mass transfer [25]. In this assay, 3 g of thawed lettuce were placed in a 30 mL polypropylene tube with a ceramic bead homogenizer before adding 6 mL of the extraction solvent. One set of samples was extracted with acetonitrile-formic acid (97:3, *v*/*v*) and the other set was extracted with acetonitrile-water (80:20, *v*/*v*). After being vortexed for 5 min, samples were sonicated 15 min and centrifuged at 4500 rpm 10 min. Thereafter, the supernatant was collected in 15 mL polypropylene tubes. Samples were re-extracted with 5 mL of the extraction solvent sonicating for 15 min. Once centrifuged, 10 min, the combined extracts were concentrated to 5 mL before a cleanup by dispersive solid-phase extraction (dSPE) with C18 (1 g). Extracts were then concentrated to 1 mL, brought to 2 mL with acetonitrile-water (20:80, *v*/*v*) and filtered through 0.22-µm nylon filters. Figure 1A shows the results of this assay.

When acetonitrile in acidic conditions was employed as solvent recoveries up to 73% were achieved for lincomycin and enrofloxacin, however for sulfonamides extraction efficiencies < 5% were obtained. In the case of employing acetonitrile-water (80:20, *v*/*v*) in the extraction, an improvement on the extraction yields of most the antibiotics was observed although for sulfonamides the recovery results did not exceed 42%. The effect of the addition of Na_2_EDTA (0.3 g) in the extraction process was evaluated and no effect in the extraction recoveries was observed.

The recovery of sulfonamides was evaluated performing the extraction of the analytes from lyophilized samples using both extraction mixtures from the previous assay. The amount of lyophilized lettuce employed in this assay was 0.2 g, which is the weight obtained after freeze-drying 3 g of frozen samples. Two sonication cycles (15 min each) were performed and the extracts were collected after 10 min centrifugation. The combined extracts were concentrated to 5 mL prior to a dSPE cleanup with C18 as in the previous assay. After cleanup, extracts were evaporated to 0.5 mL and reconstituted to 2 mL with aqueous formic acid 0.1%-acetonitrile (90:10, *v*/*v*). Figure 1B shows that similar recoveries were obtained for lincomycin, enrofloxacin and chlorotetracycline with both extraction mixtures. The extraction of sulfamethazine and sulfamethoxazole clearly improved when the sample was lyophilized and the best results were achieved when acetonitrile-water (80:20, *v*/*v*) was employed. Taking into account the results obtained in these assays, the use of lyophilized samples and acetonitrile-water (80:20, *v*/*v*) were selected as a starting point for further assays although for doxycycline and ciprofloxacin lower extraction yields were achieved.

**Figure 2:**
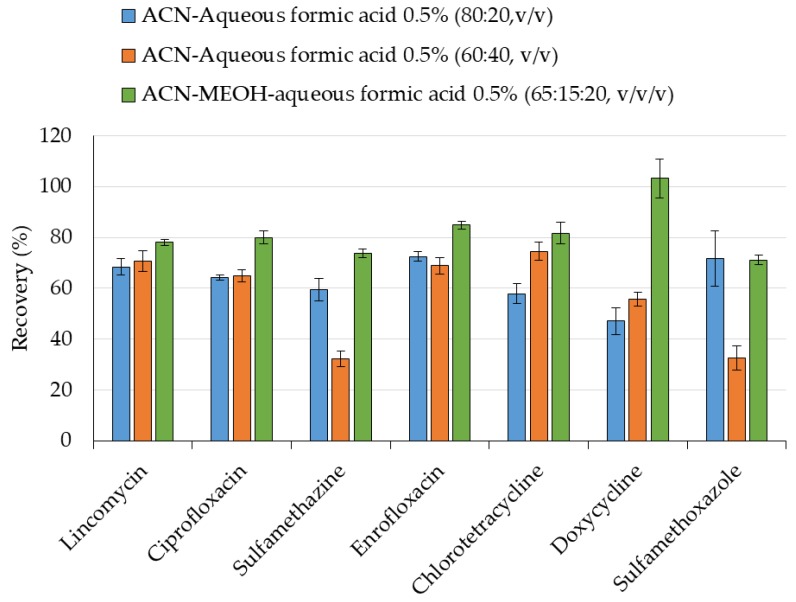
Effect of extraction solvent on the recovery of antibiotics (200 ng) from lyophilized lettuce spiked at 1000 ng/g dw (equivalent to 66 ng/g fw).

In order to improve the extraction efficiency, modifications in the extraction solvent, acetonitrile-water (80:20, *v*/*v*), were assayed, such as the extraction with aqueous formic acid instead of water, a decrease in the proportion of organic phase (acetonitrile-aqueous formic acid 0.5% (60:40, *v*/*v*), or the addition of methanol in the extraction solvent (acetonitrile-methanol-aqueous formic acid 0.5% (65:15:20, *v*/*v*/*v*). As shown in Figure 2, the addition of formic acid increased the recovery of most antibiotics except for sulfonamides that decreased especially when a lower proportion of acetonitrile was employed in the extraction. However, the addition of methanol to the extraction solution, maintaining the proportion of organic phase, provided the highest extraction efficiency for most antibiotics, particularly for doxycycline with recoveries around 100%, except for sulfamethoxazole that was slightly lower than when the extraction is carried out with acetonitrile-water (80:20, *v*/*v*).

### 2.2. Method Validation

The developed method was validated in terms of linearity, accuracy, precision, and limits of detection and quantification.

To evaluate the linearity, a set of five calibration solutions were prepared in a concentration range from 10 to 2000 ng/g dry weight (dw) employing sample extracts. Another set of calibration solutions in neat solvent was also prepared in the range from 1 to 100 ng/mL which is equivalent to the range of the previous set. Both sets were injected and a good linear response was obtained in the range of concentrations studied with correlation coefficients equal or higher than 0.991 for all the compounds. Matrix-induced signal enhancement or suppression is very common on ESI and was evaluated calculating the slope ratio between matrix-matched and solvent calibration. A suppression of the chromatographic signal was observed for all the studied antibiotics (slope ratio 0.3–0.6) except for chlorotetracycline that showed an enhancement of the signal with matrix-matched standards (slope ratio 2.0), therefore, in order to counteract these effects matrix-matched standards were used.

The accuracy of the method was evaluated performing the recovery of target analytes from lettuce spiked at two concentration levels, 50 and 1000 ng/g dw. As shown in Table 1, the recovery results obtained were satisfactory (>70%) for most of the compounds. 

The precision of the method was evaluated in terms of repeatability (intra-day precision) and reproducibility (inter-day precision). Repeatability was evaluated by analyzing six replicates within a given day and reproducibility by determining the recoveries of six replicates within different days and RSD lower than 9% and 14%, respectively, were obtained.

To evaluate the sensitivity, method detection limits (MDLs) and limits of quantification (LOQs) of the developed method were determined using eight replicates of lettuce extract, spiked at the lowest recovery level (50 ng/g dw). The MDL was determined with the following equation: MDL = SD × t_99_ where SD is the standard deviation of the replicate analysis and t_99_ is Students’ value for a 99% confidence level and n-1 degrees of freedom [26]. The LOQ was calculated as 10 times the standard deviation of the results of the replicate analysis. As shown in Table 1, the MDL values ranged from 3 to 13 ng/g dw, which are similar to those recently reported by Tadic et al. [24] for the determination of antibiotics in four types of vegetables. 

### 2.3. Application to the Uptake of Antibiotics in Lettuce

**Figure 3:**
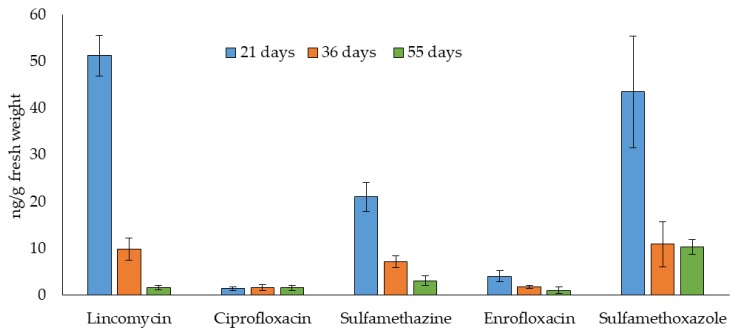
Antibiotic levels (ng/g fw) in lettuce leaves harvested 21, 36, and 55 days after planting.

The uptake of the antibiotics in lettuce shoots harvested after 21, 36, and 55 days is shown in Figure 3. Five of the seven studied antibiotics were found at quantifiable levels but tetracyclines were not detected in any of the lettuce samples analyzed. Thus, 21 days after planting, the highest antibiotics uptake was found for lincomycin (51 ng/g fresh weight (fw)), followed by sulfamethoxazole (44 ng/g fw) and sulfamethazine (21 ng/g fw). Table 2 shows the uptake factors (UF) of the studied antibiotics calculated from equation UF = Cp/Cs, where Cp is the concentration in plant material and Cs is the concentration added to soil. Our results showed uptake factors ≤ 0.02 for the studied compounds.

## 3. Discussion

The methods developed for the determination of pharmaceutical and personal care products in plant tissues usually need an additional cleaning step using SPE cartridges and the extraction methods, mainly employed for specific antibiotic compounds, required long sample preparation times and large amounts of organic solvents [20]. Some of the multiresidue methods reported showed that the recoveries for some of the antibiotics studied were low (≤60%) [6,21,24]. In the UAE method recently developed by Tadic et al. [24], the use of isotope labeled standards, as surrogate and internal standards, to correct recoveries did not increase antibiotic recoveries above 60%. 

The first assays carried out using acetonitrile in acidic conditions yielded recoveries up to 73% for lincomycin and enrofloxacin, but very low extraction efficiencies were obtained for sulfonamides. Although recoveries increased using acetonitrile–water (80:20, *v*/*v*) in the extraction, sulfonamides recoveries still remained low. The use of Na_2_EDTA solution as a chelator for multivalent cations enhances the extraction of polar compounds and it has been applied to reduce the complexation of tetracyclines with metal cations to enhance their extraction from soil and plants [6,22]. Nevertheless, we observed that, in our case, the addition of 0.3 g of Na_2_EDTA in the extraction process did not show changes in the recoveries; therefore, Na_2_EDTA was not further assayed. An improvement in the extraction of sulfamethazine and sulfamethoxazole was obtained with lyophilized samples, using aqueous formic acid instead of water and the addition of methanol in the extraction solvent. The use of a percentage of MeOH in the extraction mixture has been reported to increase the extraction of quinolones and tetracyclines [21]. In our study, a positive effect in the extraction efficiency for most antibiotics was obtained with a 15% of MeOH in the extraction mixture. Thus, the extraction mixture of acetonitrile–methanol–formic acid 0.5% (65:15:20, *v*/*v*/*v*) was selected because it provided good recoveries for all the target antibiotics, ranging from 67% to 107%.

In this study, lettuce was selected mainly because it is the most cultivated leaf vegetable in the world, and a fast-growing plant easy to grow under greenhouse conditions [27]. In addition, this product is generally consumed with minimal processing and its fibrous root structure facilitates the uptake of organic contaminants from soil [23,27]. Table 3 summarizes the antibiotics uptake studies that have been carried out using lettuce. In general, the levels found for the same antibiotic are very different. A higher uptake of lincomycin than sulfamethoxazole in the present work is in agreement with levels reported by Sallach et al. [19] for two lettuce cultivars irrigated with wastewater containing these antibiotics, although our results are in the lower end of the range. The authors observed that the lettuce cultivar had a significant influence in the uptake of antibiotics, being lincomycin levels in one cultivar up to ten times higher than in the other at the same harvest time. The differences in the uptake could be due to factors such as plant composition, root structure, root exudates, or endophyte population [19]. Harvest date was found significant for lincomycin, sulfamethazine, sulfamethoxazole and enrofloxacin levels, whereas similar values were found for ciprofloxacin in the three harvest times. The highest concentration in lettuce was found at the first harvest time (21 days); whereas, an important decrease in levels was observed for lincomycin, sulfamethoxazole and sulfamethazine after 36 days, and the concentration for all compounds was then lower or similar after 55 days (Figure 3). An explanation to the decrease in the concentration of these compounds with longer harvest times is that the plant growth rate exceeds the uptake rate or that there is a degradation or biotransformation of antibiotics in the plant. The levels found in lettuce leaves are consistent with those reported in the literature, which pointed out that lincomycin shows the highest uptake [19]. In our work, antibiotics were transferred from the amended soil to the plant, whereas in the mentioned study the antibiotics were taken up from the irrigation water. In a previous study carried out in our laboratory about the persistence and availability of antibiotics in manure–soil systems [28], it was shown that the route of entry of antibiotics into the environment, through irrigation using recycled water or through manure application, may have an important effect on their behavior. In a manure soil system, antibiotics need to be present in the soil solution to be available for plant uptake. In the mentioned study, the levels in soil solution of chlortetracycline, doxycycline, ciprofloxacin, and enrofloxacin were very low, whereas the highest levels found were for sulfamethoxazole, followed by sulfamethazine and lincomycin, which also are the antibiotics detected in lettuce leaves at the highest concentrations in the present study. 

The UF obtained for the studied compounds were ≤ 0.02, which suggests minimal uptake and accumulation potential in lettuce at environmentally relevant concentrations of antibiotics in soil amended with composted poultry manure. In agreement with these results, Sidhu et al. [29] reported an uptake factor for ciprofloxacin significantly lower than 0.01, at environmentally relevant concentrations of ciprofloxacin in biosolids-amended soils. On the other hand, taking into account the mass balance on lincomycin and sulfamethoxazole in the study by Sallach et al. [19], the highest uptake factors calculated for both substances were 0.22 and 1.64, respectively, which are significantly higher than those determined in the present work. The comparison of results in the uptake of antibiotics by lettuce is difficult because factors such as the entry route (irrigation water, nutrient solution, biosolids–soil system, or manure–soil system), lettuce varieties, cultivation, harvest time, and antibiotics concentrations applied have a significant influence in the residues found in the plant.

## 4. Materials and Methods

### 4.1. Reagents and Materials

Five antibiotics, enrofloxacin, ciprofloxacin, sulfamethazine, sulfamethoxazole and doxycycline, all with purity ≥ 98%, were purchased from Sigma (Steinheim, Germany) whereas chlortetracycline hydrochloride (purity 93.7%) and lincomycin hydrochloride (purity 100%) were supplied by Fluka (Seelze, Germany). Stock solutions were prepared at 1000 µg/mL in methanol or in methanol with 20% ammonia (for enrofloxacin and ciprofloxacin). Working standard solutions containing all analytes were prepared by an appropriate dilution of the stock standard solutions with acetonitrile. All solutions were stored in amber vials at −20 °C. HPLC grade acetonitrile and methanol were purchased from Honeywell (Seelze, Germany). Formic acid (purity ≥ 98%) was acquired from Acros Organics (Geel, Belgium). Anhydrous sodium sulfate reagent grade, extra pure sodium chloride, bulk Extrabond^®^ C18 and PSA were obtained from Scharlab (Barcelona, Spain). Microcrystalline cellulose was purchased from Merck (Darmstadt, Germany). Ultrahigh purity water was obtained from a MilliQ water purification system (Millipore, Spain).

### 4.2. Samples

Batavia lettuce (*Lactuca sativa*) seedlings were planted in pots, one in each pot with 2 kg of soil containing 50 g of composted poultry manure added as soil amendment. A total of 16 pots, considered as control samples, were placed in a greenhouse under ambient temperature conditions from April to June in Madrid, Spain. In another set of 16 pots, the composted poultry manure (50 g) was previously spiked with 5 mL of a standard solution containing all the antibiotics at 1 mg/mL and left overnight to allow the evaporation of the solvent. Then, the doped compost was added to soil and thoroughly mixed before planting the seedling. Pots were watered every 2–3 days to maintain a moderate soil moisture. Lettuces (4 replicates) were sampled after 21, 36, and 55 days of cultivation. Shoots were weighed and kept at −20 °C until lyophilization. 

### 4.3. Sample Preparation

The extraction was carried out with 0.2 g of lyophilized lettuce that were placed in a 50 mL polypropylene centrifugation tube. A 5 mL volume of acetonitrile-methanol-formic acid 0.5% (65:15:20, *v*/*v*/*v*) was added to the tube before mixing in a Multireax shaker (Heidolph Instruments GmbH & CO, Schwabach, Germany) for 5 min. Then, the samples were placed in a Branson 3800 ultrasonic bath (Emerson, St. Louis MO, USA) at room temperature for 15 min. The samples were then centrifuged at 5000 rpm for 10 min at 15 °C. After centrifugation, the extract was collected in 15 mL polypropylene tubes. The sample was sonicated again for 15 min with 5 mL of the extraction mixture and centrifuged to collect the supernatant. The combined extracts were concentrated to 5 mL using a Genevac EZ-2 evaporator (Net Interlab, Madrid, Spain). The cleanup of the concentrated extract was carried out by dispersive solid-phase extraction (dSPE) with 1 g of C18 shaking for 2 min and centrifuged for 10 min. The collected supernatant was concentrated to 0.5 mL and brought to a final volume of 2 mL with acetonitrile-formic acid 0.1% (20:80, *v*/*v*). An aliquot was filtered through 0.22 µm nylon filters.

### 4.4. LC-MS/MS Analysis

Analyses were performed on an Agilent 1200 LC system (Waldbronn, Germany) equipped with an autosampler, a quaternary pump and a column heater. A Kinetex XB-C18 (100 mm × 3 mm i.d., 2.6 µm particle size) analytical column with a C18 security guard cartridge from Phenomenex (Torrance, CA, USA) was employed for the chromatographic separation of the analytes. The column was kept at 30 °C and a flow rate of 0.3 mL/min was used. The mobile phase consisted of (A) acetonitrile and (B) 0.1% formic acid in water with the following gradient program: 0 min, 95% B; 10 min, 70% B; 12 min, 60% B; 13 min, 20% B; 16 min, 20% B; 17 min, 95% B; 20 min, 95% B.

Mass spectrometry was performed with an Agilent 6410 triple quadrupole mass spectrometer equipped with an electrospray ionization (ESI) interface, operating in positive ion mode. The following mass spectrometer parameters were set: Drying gas temperature of 300 °C, drying gas flow rate of 9 L/min; nebulizer gas pressure of 45 psi and capillary voltage of 4000 V.

For both identification and quantification of the analytes, one precursor ion and two product ions for each target compound were selected to work in multiple reaction monitoring (MRM) mode. The precursor and product ions with their optimal collision energies and fragmentor voltages, are summarized in Table 4. Analytes were confirmed by their retention time and the identification of quantifier and qualifier transitions. Retention times must be within ± 0.2 min of the expected time and qualifier-to-quantifier ratios within a 20% range for positive confirmation. 

## 5. Conclusions

A method, based on UAE and dSPE clean-up, was successfully developed for the analysis of antibiotics, belonging to different classes, in lettuce. The method showed satisfactory recovery values for all antibiotics, and LODs between 3 and 13 ng/g dw. After validation of the method, the procedure was applied to determine the uptake of these compounds by lettuce grown in a soil–manure system and harvested at three different times (21, 36, and 55 days). All samples showed uptake of five of the antibiotics evaluated; whereas both tetracyclines were not detected in lettuce leaves. The highest concentrations were obtained 21 days after planting and the highest antibiotics uptake was found for lincomycin, followed by sulfamethoxazole and sulfamethazine. Residual concentrations of antibiotics in wastewater, biosolids or manure could be a source of antibiotics in soil. Thus, during the growing period a vegetable crop can take up those antibiotic residues that could be present in the consumer product, particularly in vegetables consumed with minimal processing, such as lettuce. Although the levels found were low, the presence of antibiotics in lettuce demonstrates the need for further assessing food safety risks related with the use of soil amendments or irrigation water contaminated with antibiotics.

## Figures and Tables

**Table 1:** Recoveries and relative standard deviations (*n* = 4) obtained for the target antibiotics from lettuce spiked at two concentrations. Method detection limits (MDL) and limits of quantification (LOQ) obtained for the selected analytes (expressed in ng/g dw).

	1000 ng/g dw		50 ng/g dw		MDL	LOQ
	Mean (%)	RSD (%)	Mean (%)	RSD (%)		
Lincomycin	78	1	83	3	3	10
Ciprofloxacin	80	3	90	6	7	24
Sulfamethazine	74	2	67	5	10	33
Enrofloxacin	85	2	70	9	3	10
Chlorotetracycline	82	4	93	8	13	45
Doxycycline	107	8	79	6	12	38
Sulfamethoxazole	71	2	73	6	5	15

**Table 2:** Uptake factor (UF) of the antibiotics after 21, 36, and 55 days of growth. (Uptake factor values were calculated from equation: UF = CP/CS, where Cp is the concentration in plant material and Cs is the concentration added to soil).

Days	Lincomycin	Ciprofloxacin	Sulfamethazine	Enrofloxacin	Sulfamethoxazole
21	0.020	0.001	0.008	0.002	0.017
36	0.004	0.001	0.003	0.001	0.004
55	0.001	0.001	0.001	0.0004	0.004

**Table 3:** Summary of studies where the uptake of antibiotics by lettuce was evaluated.

Analytes (Compounds in Common with the Present Work)	Lettuce Variety	Spiking Concentration	Antibiotic Entry Route	Harvest Time (days)	Conditions	Antibiotic Uptake by Lettuce Leaves	Ref.
10 veterinary medicines (ENR)	All Year Round (Butterhead)	1 mg/kg soil	Soil	103	70% humidity, 20 °C light cycle 15 °C dark cycle	ENR: < LOD	[5]
SFZ	Not stated	50 and 100 mg/L manure (1.25 and 2.5 mg/kg soil)	Soil–manure system	45	Greenhouse 18–23 °C	SFZ: 1000–1100 µg/kg dw	[7]
11 pharmaceuticals (LIN, SFX)	Black Seeded Simpson	50 and 30 μg/L	Irrigation water	7, 14, 35	Greenhouse, 24 °C 43% humidity	LIN: 1–6 µg/kg fw SFX: 0.05 µg/kg fw	[13]
6 antibiotics (CTC, SMX, SMZ)	Not stated	5, 10 and 20 mg/kg soil	Irrigation water	45	Greenhouse, 25 °C 70% humidity	TCs: 10–200 µg/kg SFs: 400–3200 µg/kg	[14]
7 antibiotics (LIN, SFX)	Green Star Salad Bowl	1 mg/L water	Irrigation water	24, 35, 46	Greenhouse 15–18 °C	LIN: 84–822 µg/kg fw SFX: 22–125 µg/kg fw	[19]
2 antibiotics (CIP)	Buttercrunch	0.01, 0.115, 0.371 mg/kg soil	Soil-biosolids system	46	Greenhouse 20–25 °C	CIP: 4–5 µg/kg dw	[29]
19 PPCPs (SFX)	Iceberg	500 ng/L	Nutrient solution	21	Greenhouse, 12–32 °C 40–90% humidity	SFX: <LOD	[30]
7 antibiotics (CIP, CTC, DOX, ENR, LIN, SMX, SMZ)	Batavia	2.5 mg/kg soil	Soil-poultry manure system	21, 36, 55	Greenhouse ambient conditions	FQs: 1–4 µg/kg fw TCs: < LODs SFs: 3–43 µg/kg fw LIN: 2–51 µg/kg fw	Present work

CIP: ciprofloxacin, CTC: Chlortetracycline, dw: dry weight, ENR: enrofloxacin, fw: fresh weight, LIN: lincomycin, PPCPs: pharmaceutical and personal care products, SFX: sulfamethoxazole, SFZ: sulfamethazine, TCs: tetracyclines.

**Table 4:** Optimized multiple reaction monitoring (MRM) conditions for the analysis of the selected antibiotics.

Compound	MRM 1	CE (eV)	MRM 2	CE (eV)	Fragmentor (V)
Lincomycin	407 > 359	15	407 > 126	30	150
Ciprofloxacin	332 > 314	18	332 > 231	42	130
Enrofloxacin	360 > 342	18	360 > 316	18	130
Chlortetracycline	479 > 462	15	479 > 444	20	120
Doxycycline	445 > 428	25	445 > 154	30	120
Sulfamethazine	279 > 186	15	279 > 124	20	130
Sulfamethoxazole	254 > 156	15	254 > 92	25	100

CE = collision energy.
